# Does severe hyperlactatemia during cardiopulmonary bypass predict a worse outcome?

**DOI:** 10.1016/j.amsu.2021.103198

**Published:** 2021-12-21

**Authors:** Aniss Seghrouchni, Noureddine Atmani, Younes Moutakiallah, Abdelkader Belmekki, Youssef El Bekkali, Mahdi Ait Houssa

**Affiliations:** a Cardiovascular Surgery Department, Mohammed V Military Training Hospital, Rabat, Morocco; b Faculty of Medicine and Pharmacy, University of Sidi Mohamed Ben Abdellah, Fes, Morocco; c Hematology Teaching and Research Unit, Faculty of Medicine and Pharmacy, Rabat, Morocco

**Keywords:** Cardiac surgery, Cardiopulmonary bypass, Hyperlactatemia, Postoperative complications

## Abstract

**Introduction:**

The aim of the current study was to evaluate the impact of increased blood lactate levels during cardiopulmonary bypass (CPB) on immediate results in patients who underwent open heart surgery.

**Materials and methods:**

We performed a retrospective single-center study on 1290 patients. Adult cardiac surgical patients who underwent valve surgery, coronary artery bypass graft, combined procedure, adult congenital anomalies and aortic surgery were enrolled. Patients with associated comorbidities such as liver dysfunction, hemodynamic instability before surgery were excluded. Arterial blood lactate concentration was measured immediately after weaning from CPB and evaluated together with clinical data and outcomes including in hospital mortality. Patients were classified into 3 groups according to their peak arterial lactate level: group I [normal lactatemia, lactate ˂ 2 mmol/l (n = 749)], group II [mild hyperlactatemia, lactate 2–5 mmol/l (n = 489)], group III [severe hyperlactatemia, lactate ˃ 5 mmol/l (n = 52)]

**Results:**

When comparing outcomes across the 3 groups, severe hyperlactatemia was correlated with worse outcomes including higher in-hospital mortality, low output cardiac syndrome, postoperative renal insufficiency, myocardial infarction, red blood cell transfusion (RBC) transfusion, prolonged mechanical ventilation and longer intensive care unit (ICU) stay hours.

**Conclusion:**

Blood lactate level above 5 mmol/l and more during CPB is associated with higher in-hospital mortality rate and postoperative complications. More attention must be given to correct the common abnormalities conditions inherent of CPB in order to conduct adequate tissue perfusion and reduce the risk of hyperlactatemia.

## Highlights

• Lactatemia reflects the state of organ functions.
• Lactate concentration is a potential biomarker in patients treated with cardiac surgery.
• Blood lactate level > 5 mmol/L at the end of CPB is a risk factor of pooroutcomes.
• Closer follow up is recommended to preserve accurate tissue perfusion to improve prognosis.

## 1 Introduction

The current therapeutic management of human diseases is performed based on global patients’ parameters of hemodynamic and metabolic status including lactate concentration as a biomarker of outcomes [1,2]. Recently, this prognostic predictor has been incorporated into an important number of postoperative scores [[3], [4], [5], [6], [7]]. As mentioned in the literature, blood lactate level reflects important vital patterns including liver and respiratory functions, cardiac function, venous saturation, catecholamine administration and hyperglycemia [[8], [9], [10], [11]]. Cardiopulmonary bypass (CPB) is an unphysiological state that could disturb tissue perfusion and lead to increased lactate levels. Elevated lactate level is associated with inferior prognostic outcomes in many conditions and diseases [12]. In cardiac surgery, several reports found a strong correlation between elevated perioperative lactate concentration and postoperative adverse events such as mortality [5,[13], [14], [15]]. In this real-world cohort, we investigated the hypothesis that increased lactatemia within the end of CPB could predict immediate complications and in-hospital mortality.

## 2 Patients and methods

### 2.1 Enrollment and inclusion criteria

This was a retrospective observational study that was performed at the authors' institution. Data from all adult patients (>18 years) undergoing cardiac surgery over a 19-year period, between January 2000 and December 2018 were included and analyzed. The study was approved by the local ethics committee. Patient's characteristics and perioperative data were obtained from the institutional database. Patients were excluded if the chart entries were incomplete, blood lactate level was not measured, or surgery involving deep hypothermic circulatory arrest. Moreover, patients with factors predisposing to lactic acidosis such as metformin, anti-retroviral drugs, isoniazid, septic shock were also excluded. Among 2590 patients who underwent cardiac surgery in our institution, 1284 patients were finally included in the study.

The risk of surgical complications was predicted using EuroSCORE (European System for Cardiac Operative Risk Evaluation). Before the intervention, all enrolled patients received a usual premedication and treated according to the cardiac anesthesia protocol. Cardiopulmonary bypass was established in a standardized manner. The CPB circuit consisted of a roller pump in a non-pulsatile mode. All surgeries were performed via median sternotomy. CPB was conducted with moderate hypothermia (32–34 °C). The heart was arrested with aortic cross clamping and infusion of anterograde cardioplegia. Myocardial protection was performed by blood cold cardioplegia. Flow rates were adjusted to maintain mean arterial blood pressure between 50 and 70 mmHg. Patients were weaned from CPB in a conventional fashion. In the intensive care unit (ICU), postoperative care for our patients after surgery was standardized and accomplished according to our local institutional postoperative surgery protocol. Blood lactate parameters as well as arterial blood gases (ABGs) were measured routinely after anesthesia induction and initiation of CPB. According to the peak arterial blood level measured immediately after weaning from CPB, patients were classified into three groups:

Group 1: lactate level ˂ 2 mmol/L

Group 2: lactate level between 2 and 5 mmol/L

Group 3: lactate level >5 mmol/L [16].

The primary outcome was mortality, defined as all causes of death within 30 days after surgery. Secondary outcomes included the following postoperative complications: low output cardiac syndrome (LOCS), postoperative renal insufficiency (RI), respiratory complications, neurologic events, re-exploration for bleeding, sepsis, perioperative and myocardial infarction (MI).

### 2.2 Statistical analysis

Patients' data were collected and analyzed using SPSS software version 19.0 (SPSS In, Chicago Illinois). Quantitative data were presented as means with their standard deviations (SD) or median with their interquartile ranges and categorical data as numbers or relative frequencies. Statistical differences among groups were evaluated using the analysis of variance (ANOVA). When data were normally distributed, groups were compared using unpaired Student's *t*-test. Non-parametric tests such as Mann-Whitney U or Kruskal Wallis tests were utilized to compare non-normally distributed variables. The correlation between increased lactate level and morbi-mortality was investigated by Pearson's test. Also, the sensitivity and specificity of blood lactate concentration as a predictor of worse outcome were assessed using the receiver operating characteristic curve (ROC). For all the tests, a p value of ˂ 0.05 was considered as significant. The results of our study were reported according to the STROCSS criteria [[1], [2], [17]].

## 3 Results

2590 adult patients who underwent cardiac surgery with CPB between 2000 and December 2018 at the author's institution were identified. A lactate measurement was not obtained in 1300 of the patients after weaning from CPB. Finally, 1290 patients were enrolled in the present study. 749 patients had a peak arterial lactate level ˂ 2 mmol/l (1.5 ± 0.4), 489 patients had a peak arterial lactate level between 2 and 5 mmol/l (2.94 ± 0.6) and 52 patients had a peak arterial lactate level ˃ 5 mmol/l (6.46 ± 2).

Preoperative profile data of the patients are reported in Table 1. As shown, the three groups were comparable in terms of age and female gender. The prevalence of cardiovascular risk factors (diabetes mellitus, smoking, dyslipidemia, hypertension, obesity), are similar among the three groups. All patients were symptomatic at the time of surgery, but patients with a peak arterial lactate level ˃ 5 mmol/l were more symptomatic compared to other groups (p = 0.042). Also, the incidence of comorbidities (Renal insufficiency, Chronic obstructive pulmonary disease COPD, anemia) were similar between groups. However, patients with a peak arterial lactate level more than 5 mmol/l had more anemia than other groups (p = 0.011).

**Table 1:** Preoperative profile data of the patients.

Variable	GI (n = 749)	GII (n = 489)	GIII (n = 52)	P value
Age (years)	53.8 ± 13.8	54.4 ± 12.4	53.4 ± 12.5	0.67
Female gender (%)	37%	39%	40.3%	0.71
BMI (Kg/m^2^)	25.4 ± 4.3	25.6 ± 3.8	26.4 ± 4.3	0.19
Redo cardiac surgery (%)	6	9.2	7.9	0.11
Diabetes mellitus (%)	24	29.8	25	0.07
Hypertension (%)	25.3	26.7	25	0.83
Smoking (%)	38	36.3	36.5	0.79
NYHA III-IV (%)	41.8	42.1	59.6	0.042
AF (%)	23.7	27.4	32.7	0.16
Anemia (%)	25.9	18.8	29	0.011
COPD (%)	6.5	6.5	7.6	0.95
Renal insufficiency (%)	4.7	4.7	9.6	0.27
Valvular disease (%)	57.4	54.6	57.7	0.59
Coronary disease (%)	38	40.3	28.8	0.24
Coronary + valvular disease (%)	4.8	5.2	3.8	0.93
LVEF ≤40% (%)	11.9	10.6	15.3	0.52
PASP ≥60 mmHg (%)	24.6	22.4	42	0.055
Euroscore	2.8 ± 2.4	3.1 ± 3	4.1 ± 3.3	0.002
Multivalvular disease (%)	23.8	29.7	36.5	0.004

Abbreviations: BMI: body mass index; AF: atrial fibrillation; COPD: chronic obstructive pulmonary disease; LVEF: left ventricle ejection fraction; PASP: pulmonary artery systolic pressure.

No differences were observed between groups regarding types of heart disease (valvular, coronary, combined procedures). However, multivalvular heart disease was frequent in patients who experienced a peak arterial level lactate more than 5 mmol/l (p = 0.044) and had increased pulmonary arterial pressure (p = 0.055). Table 2 shows the perioperative data of the three patients’ groups. We observed a significant association between the increase in lactate level during CPB and the mortality rate, when the peak of arterial lactate level was ˃ 2 mmol/l, the mortality rate was 3 times higher than in group I (lactate level ˂ 2 mmol/l). In addition, one patient among 4 died when the peak arterial level was ˃ 5 mmol/l (p = 0.0001).

**Table 2:** Perioperative data of the three patients’ groups.

Variables	GI (n = 749)	GII (n = 489)	GIII (n = 52)	P value
No elective surgery (%)	4	5.5	21	0.0001
CPB time (min)	114.3 ± 41	124.8 ± 45.6	152.3 ± 62	0.0001
CPB time ˃ 120 min (%)	42.4	50.8	63.4	0.0001
Aortic cross clamping (min)	80 ± 33.9	83.3 ± 36	102.7 ± 55	0.0001
**Surgical procedure time (min)**				
Mechanical ventilation time (Hours)	6 (4–14)	7 (5–17)	18 (9–72)	0.0001
Mechanical ventilation ˃ 48 H (%)	4.9	11	36.5	0.0001
ICU stay (Hours)	48 (40–66)	48 (44–72)	72 (48–144)	0.0001
Hospital stay (days)	11 (9–14)	11 (10–15)	12 (9–14)	0.0001
Need of inotropic drug (%)	9.5	15.9	44.2	0.0001
IABP (%)	4.9	8.6	23	0.0001
LOCS (%)	7.2	13	30.7	0.0001
Renal insufficiency (%)	5.5	8.6	30.7	0.0001
Dialysis (%)	0.8	2.4	5.8	0.005
Reexploration for bleeding (%)	3.7	3.9	3.8	0.99
RBC transfusion (%)	30.4	38	51.9	0.001
GI complications (%)	1.3	1.02	5.8	0.02
Pulmonary infection (%)	9.5	10	17.3	0.19
CVA (%)	0.6	1	1.9	0.56
MOF (%)	2.3	5.9	2.3	0.0001
30-day mortality (%)	3.1	8.8	25	0.0001
Lactate level	1.5 ± 0.4	2.94 ± 0.6	6.46 ± 2	0.0001
AMI (%)	1.6	7.7	15.3	0.0001

Abbreviations: CPB: Cardiopulmonary bypass, ICU: Intensive care unit, IABP: Intra-aortic balloon pump, LOCS: low output cardiac syndrome, RBC transfusion: Red blood cell transfusion, GI complications: Gastro-intestinal complications, CVA: Cerebro-vascular accident, MOF: Multi organ failure, AMI: Acute myocardial infarction.

**Fig. 1:**
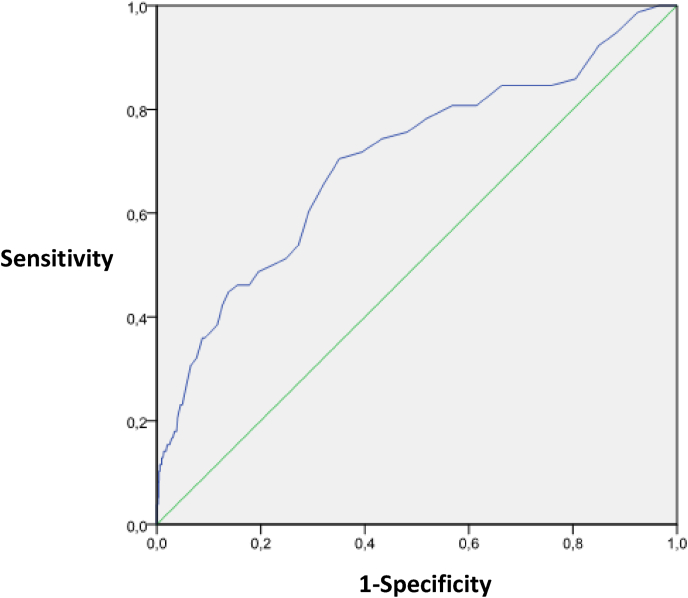
Receiver operating characteristic (ROC) curve of optimal lactate level as a predictor of mortality.

We noted a significant correlation between intra-operative increase in the lactate level and immediate worse outcome. Patients in group III, whose intra operative lactate level increased demonstrated postoperative LOCS (30.7%, p = 0.0001), had more postoperative renal insufficiency (30.7%, p = 0.0001), half of the patients needed RBC transfusion (51.9%, p = 0.001), and developed frequently gastro intestinal (GI) complications (5.8%, p = 0.02). These patients compared with those who experienced an intraoperative no increase in the lactate level (group I and II) had the longest ICU stay 72 (48–144) hours (p = 0.0001). The duration on CPB was correlated with a change in lactate level (p = 0.0001). Fig. 1 shows the ROC curve of optimal lactate level as a predictor of mortality. A peak arterial lactate level of 5 mmol/l showed good discrimination between the outcomes groups, with an area under the curve (AUC) is 0.74 (95% CI: 0.62–0.76). We noted significant correlation between CPB duration timing surgery and elevated blood lactate concentration (r = 0.19, p = 0.0001; r = 0.19, p = 0.0001 respectively). A similar correlation was found between increased lactate level and hospital death, mechanical ventilation time, and ICU stay: (r = 0.38, p = 0.005), (r = 0.16, p = 00001), (r = 0.11, p = 0.0001), respectively.

## 4 Discussion

Tissue perfusion during cardiopulmonary bypass can be monitored by different biomarkers. Blood lactate levels are the most used biomarker that may reflect adequate perfusion during cardiac surgery. The association between hyperlactatemia and outcomes has been reported in several studies [7,^14^,[1], [2], [17],18]. This was confirmed in the present retrospective study that found that an increase in the lactate level during CPB is significantly associated with mortality and postoperative complications.

A peak arterial lactate level above 5 mmol/L in the end of CPB has been correlated with higher mortality, 8- fold when compared with those who had normal lactate level (3.1% vs 25%, p = 0.0001). Another important finding was that patients with a peak lactate level ˃ 5 mmol/l developed LOCS frequently, need more inotropic drugs and intra-aortic balloon pump (IABP). Additionally, renal insufficiency, prolonged mechanical ventilation and longer length of ICU stay were more prevalent. These findings are in line with available literature [[19], [20], [21], [22], [23], [24], [25], [26], [27]].

There is still controversy about the timing and the frequency of blood sampling for lactate assessment during and after cardiac surgery. A number of reports have shown that the study of a single perioperative lactate level is not a useful prognostic biomarker when used in isolation [21,28]. In our study, we have measured only the lactate level at the end of CPB in order to assess the quality of tissue perfusion during CPB and its impact on immediate results.

Hyperlactatemia is a familiar finding after cardiac surgery. It occurs during or soon after CPB in 10–26.6% of patients [14,[1], [2], [17],^29^,30]. In our study, severe hyperlactatemia ˃ 5 mmol/L was observed in 7.7% of enrolled patients. This prevalence was also reported by Rannuci et al. [[1], [2], [17]]. It has been termed early hyperlactatemia while late onset hyperlactatemia develops within 6–12 h of admission to the ICU [31]. Most reports suggested that early onset hyperlactatemia is associated with poor outcomes including increased morbidity and mortality [[30], [31], [32], [33]].

In the present study population, peak arterial lactate levels above 5 mmol/l occurring from the onset of CPB to arrival in the ICU substantially increased the risk for mortality (25%). These findings are in line with the reports of several teams; for example, Demers et al. and Maillet et al. showed that, when compared with those of a normal lactatemia, patients with immediate hyperlactatemia have an eight-to-ten-fold increase in postoperative mortality [20,30].

Hyperlactatemia is common in critical conditions. Despite its etiopathogenesis is complex, it is well accepted that accumulating in the blood circulation is due to hypoperfusion of body tissues and also cell hypoxia during anaerobic metabolism [34]. Hyperlactatemia has multifactorial origins which vary between patients [12,33,35].

The literature on organ specific release during cardiac surgery is vast. Myocardial muscle has been shown to be a source of lactate during CPB [[36], [37], [38]]. It has been also demonstrated that inadequate myocardial protection could lead to myocardial damage and elevated lactate early after weaning from CPB [39,40]. In our study, we have noted a significantly higher incidence of perioperative myocardial infarction in patients who developed severe immediate hyperlactatemia (15.5%, p = 0.0001).

Skeletal muscle is also a potential source of lactate production during CPB. Recently, Dedichen et al. and Mandak et al. have assessed the effect of CABG with pump on muscle perfusion [41,42]. Their findings showed that an elevated arterial lactate concentration earlier after surgery, which was associated with an increased anaerobic metabolism in skeletal muscle. There is still a controversy about the contribution of the lungs to hyperlactatemia. However, two recent studies confirmed that the lungs are an important source of lactate production following CPB but its release may occur later, 6 h after surgical interventions [43,44].

The splanchnic circulation was reported to be associated with lactate production during cardiac surgery by various authors [[45], [46], [47]]. In our cohort, we noted more gastrointestinal complications in the postoperative period in patients experiencing severe hyperlactatemia when compared to other groups (p = 0.0011). In addition, early onset hyperlactatemia has been reported to be associated with greatly increased likelihood of adverse outcomes [21,24,33,35,48]. In our study, severe hyperlactatemia (˃ 5 mmol/l) developing during CPB was associated with poor outcomes including: higher mortality rate, higher inotropic use, higher IABP usage, more LOCS, prolonged mechanical ventilation, more renal insufficiency, higher incidence of perioperative myocardial infarction and longer duration of ICU stay. These findings are in accordance with the majority of studies reported recently [21,24,31,33,48,49]. However, recently contrary to published literature, Kim et al. did not find significant association between elevated postoperative lactate levels and mortality, acute renal insufficiency or LOCS [19].

It is well known that oxygen delivery in physiological conditions is dependent on the hemoglobin level, arterial oxygen saturation and cardiac output. To date, few reports that linked oxygen delivery on CPB with early postoperative hyperlactatemia were published. However, it is well recognized that low hemoglobin level is a potential cause of lactate production during CPB [12,18,20,21,50]. Recently, Ranucci et al. noticed that severe hemodilution on CPB might be deleterious and determinant of early postoperative hyperlactatemia [18]. During cardiac surgery, many factors lead to decrease the hematocrit value (blood loss, prime fluids, crystalloid cardioplegia). Indeed, to keep hemoglobin level correct (˃ 7 g/dl), the perfusionist must transfuse the patient or do hemofiltration [[51], [52], [53]]. Some authors suggested that the use of hemofiltration induce hemoconcentration and elevation of hematocrit and minimize blood transfusion [54]. However, Soliman et al. have shown that if doing the whole time of CPB should increase the serum lactate, and it should be limited for high-risk patients: CPB ˃ 2h, poor left ventricle ejection fraction (LVEF), impaired renal function [53]. In the current study, preoperative anemia was more prevalent in patients with severe hyperlactatemia and more of them have received RBC transfusion in the postoperative period (ICU). Recently, lactate concentration was found to represent the balance between lactate production and lactate clearance [55]. But in case of type A cardiac surgery, recognizing a hypoxic nature is dominant pattern [1], [2], [17],30]. This previous opinion is in agreement with the results published by Lopez-Delgado, who found higher lactate production in non survivors as compared to survivors [49].

Importantly, postoperative hyperlactatemia during cardiac surgery was not found to be only caused by an impairment of tissue perfusion, but it may be affected by several other factors [12,33,35,56]. Notably, CPB duration is the most important factor that is consistently implicated as a key determinant of intraoperative lactate production [20,24,48,57]. Ranucci et al. have identified a cutoff value of 96 min as predictive of hyperlactatemia during CPB [[1], [2], [17]]. Our study findings have replicated these findings. Both CPB and aortic cross clamping times were significantly longer in patients experiencing severe hyperlactatemia (152.3 ± 62 min) and (1027 ± 55 min) respectively and 63.4% of them had CPB time ˃ 120 min.

Hyperlactatemia is almost invariably associated with poor glycemic control both intraoperatively and postoperatively [1], [2], [17],^29^,^58^,59]. Theoretically, Ringer's solution used as the pump prime could increase circulating lactate levels, but several authors have compared Ringer's lactate and Ringer's acetate and noted that hemodynamic profiles and the evaluation of acid-base parameters similar were between groups [[60], [61], [62]].

Blood lactate as a prognostic marker has been used in the construction of several postoperative prediction scores [[5], [6], [7],[63], [64], [65]]. However, its accuracy depends on several parameters like specific patient populations, timing of the lactate measurement and the cutoff value used. An optimal cutoff value for assessing mortality risk was as yet unknown and may differ in different studies [66]. Our results are in line with various reports and demonstrated that an increased intraoperative lactate is strongly correlated with higher mortality and morbidity.

Mild hyperlactatemia was previously reported to be usually associated with benign conditions [67,68]. A higher value (˃ 5 mmol/L) would have slightly increased the prognostic power. Basaran et al. found that lactate levels more than 4.8 mmol/l were associated with increased mortality rates [69]. In a recent study, Haanschoten et al. found that postoperative peak arterial lactate levels above 5 mmol/L were substantially associated with increased mortality risk [21]. Moreover, Lenkin et al. showed that peak lactate concentration was 6.75 mmol/L in patients with postoperative complications [70].

Contrary to several studies supporting the use of serial lactate measurement [5,21,29,48,71,72], the authors arbitrary choose a single lactate measurement, because the main objective of this study was to assess the quality of perfusion during CPB and its impact on immediate outcomes. Consequently, other potential causes of hyperlactatemia that occurs after in ICU were excluded.

The most important limitation of the current work is its single real world observational nature. Another important factor is the long duration of study period and this parameter might have an impact on various data, specifically because of the progressive and real improvement accomplished in terms of diagnosis and treatment. Moreover, it is difficult to elucidate the link between hyperlactatemia and postoperative low cardiac output syndrome. It could be a consequence of LOCS and not itself a cause of LOCS. Perioperative management of blood pressure and pump flow vary among perfusionists and different institutional protocols including the use of vasoactive drugs (epinephrine) at the discretion in order to keep mean arterial pressure stable. Therefore, the impact of this medication on lactate production in our study is not clear. The percentage of severe hyprelactatemia was less compared to moderate hyperlactatemia, and the statistical power analysis linking increased blood lactate and worse outcome was not permissible. The authors missed some intraoperative parameters that might increase blood lactate levels during CPB. There is no doubt that blood glucose control in the perioperative period is important, unfortunately this factor was not identified. Serum lactate measurements were not performed in a standardized manner in all treated patients which is another limitation of our present work. In addition, we did not measure pre CPB lactate concentration as a reference because some patients could already have abnormal values of lactate level because of others reasons. Finally, some patients normalize their lactate level in the postoperative period and their prognosis was well. Also, it is well demonstrated that persistent hyperlactatemia is a more important risk factor than transient hyperlactatemia or lactate concentration itself. When reviewing the emerging evidence on this topic [73], it is noticed that this biomarker merits further investigation in well conducted studies to confirm its association with outcomes in this setting. We expect to improve the quality of these data and therefore our findings in the future based on prospective enrollment and prior hypothesis testing.

## 5 Conclusion

Blood lactate level more than 5 mmol/L at the end of CPB is associated with worse outcomes. Various mechanisms occurring before and during CPB may train to early postoperative hyperlactatemia and these need closer surveillance in order to keep adequate tissue perfusion and thereby improve prognosis.

## Provenance and peer review

Not commissioned, externally peer-reviewed.

## Ethical approval

The study was approved by the local ethic committee of Mohammed V Training Hospital. Data were anonymously registered in our database.

## Informed consent

Given the retrospective nature of this study, no consents were needed.

## Sources of funding

No funding was received for this research.

## Author contribution

Conceptualization: MAH and AS. Data curation: MAH and AS. Supervision: YEB and MAH. Validation: AS, NA, YM, AB. Writing: AS. All authors read and approved the final version of the manuscript.

## Research registration

Research registry7279.

## Guarantor

Dr. Aniss Seghrouchni (MD)

## Declaration of competing interest

The authors report no conflicts of interest
